# Supplementary material for: Insecticide resistance management strategies for public health control of mosquitoes exhibiting polygenic resistance: A comparison of sequences, rotations, and mixtures
Source: Evol Appl. 2023 Apr 5;16(4):936–59. doi: 10.1111/eva.13546 (PMC10130562; doi:10.1111/eva.13546)
Supplement: Supplementary file 1 — Appendix S1 [file EVA-16-936-s003.pdf]

Supplement 1: Additional Model Rationale:

Here we provide additional context for our rationale behind the model methods. The sub-headings refer to the same sub-headings as in the main manuscript methods section.

Developing a quantitative scale of resistance:

IR has many different definitions including survival in bioassays (WHO cylinder or CDC bottle), experimental huts, etc and there is no single clearly-defined scale that can be used to describe a "resistance phenotype" because this depends on the how the phenotype is measured. For example, survival in a bioassay may differ from survival in an experimental hut or insecticide sprayed wall in a house. For many quantitative traits this is a trivial process, where the trait of interest also corresponds to the amount of something produced, especially in agriculture and selective breeding where quantitative genetics is frequently leveraged. In contrast, resistance to insecticides is generally measured as a binary outcome as individuals either survive or die exposure.

The Response to Selection:

The value of β was calculated empirically so that, on average, insecticides under continuous usage would have an "insecticide lifespan" of approximately 100 mosquito generations (~10 years) in the absence of fitness costs or migration. We define "insecticide lifespan" as the time to the withdrawal threshold of 10% bioassay survival. This was achieved by sampling the parameter space of the female insecticide exposure (x), male insecticide exposure (m) and heritability (h^2) to calculate values of the response (R) using equation 4. The value of β was changed until this distribution centred around R=1, which was found to be β=10 (Figure 2). This is the critical part that calibrates/validates our PRS scale of z. It means that if z=0 at the start of the

simulation (i.e., IR is absent) it should usually reach a value of z=100 (giving 10% bioassay survival, see Equation 1(a)) after around 10 years of continuous deployment. The value of 10 years was selected as a reasonable time, based on experience (and expectation) that insecticides take roughly 10 years (i.e., ~100 generations) of deployment before starting to fail (a >10% survival rate is regarded as indicative of potential for future failure (WHO, 2018)). It is, however, user-defined, and the simulations could be recalibrated by changing β so that the “insecticide lifespan” is reached (i.e., 10% bioassay survival) most frequently occurs after 5, 15, 20 years of deployment according to operator beliefs on likely timescales and the threshold at which insecticide withdrawal should occur.

Fitness Costs:

The simplest way of incorporating a fitness cost would be to set it as an absolute value defined as the reduction in z to be applied each generation. However, the same absolute decrease in z could be sufficient to prevent the evolution of resistance in simulations where selection is weak or could have only a marginal impact in simulations where selection is very strong.

Importantly any given value of ψ now has the same impact in all runs when simulating over numerous parameter combinations, making its effect constant and hence its impact can be better estimated. $\Delta_c\bar{z}$ is the change in the mean PRS per generation as a result of the fitness costs associated with the resistance genes (ψ), noting that is trivial to set $\Delta_c\bar{z} = 0$ if costs are assumed to be absent. We first calculate the response as though the insecticide was present (in the intervention site), before multiplying this response value by the fitness costs. This helps to ensure the fitness cost is smaller than the response allowing resistance to take-off when z is small.

Special Case 1: Cross resistance and cross-selection between insecticides:

As all the traits measured in this model are on the same scale (e.g., z=100 for trait I is 10% bioassay survival to insecticide i, and z=100 for trait J is 10% bioassay survival to insecticide j), there is no need for regression/variance coefficients to translate between different scales as would arise if, for example, finding the degree of cross selection between selection of wing length (mm) and body mass (mg) as the two traits are on different scales), and we can assume a simple correlation between the traits. For example, if the genetic correlation is 10% between trait I and trait J, then insecticide selection causing a one-unit increase in z_I would cause a 0.1 unit increase in z_J.

Special Case 3: Cross resistance and Selection with Mixtures:

Our methodology can be extended to track more than two insecticides so it would technically be possible for the tracked insecticide to not be deployed in the currently deployed mixture. This would occur if insecticide i is being tracked, while a mixture containing insecticide j and k was deployed. In this instance there is indirect selection on trait I from insecticide j and insecticide k. There is therefore indirect selection on trait I from insecticide j scaled by survival to insecticide k. There is also indirect selection on trait I from insecticide k scaled by survival to insecticide j.

$$\bar{z}_t^{I'} = (\bar{z}_t^I - \psi^i R^i) + (K_k^F \alpha_{JI}(R^j - \psi^j R^j)) + (K_j^F \alpha_{KI}(R^k - \psi^k R^k))$$

Equation 8(a(ii))

Note, in our simulations, we only use two insecticides, therefore all mixture simulations use only equation 7a(iii), however equation 8a(ii) is presented for completeness and demonstrates this modelling framework can extend to allow simulations with more than two insecticides.

WHO. (2018). Test procedures for insecticide resistance monitoring in malaria vector mosquitoes (Second edition) (Updated June 2018). World Health Organisation. http://www.who.int/malaria/publications/atoz/9789241511575/en/
