# Supplementary material for: Insecticide resistance management strategies for public health control of mosquitoes exhibiting polygenic resistance: A comparison of sequences, rotations, and mixtures
Source: Evol Appl. 2023 Apr 5;16(4):936–59. doi: 10.1111/eva.13546 (PMC10130562; doi:10.1111/eva.13546)
Supplement: Supplementary file 2 — Appendix S2 [file EVA-16-936-s004.pdf]

Supplement 2: Linear modelling between bioassay survival and field survival.

Bioassay Survival and Field Survival Conversion

The model tracks the bioassay survival to insecticides via the PRS (Equation 1a). Laboratory bioassays are highly standardised using unfed, 3–5-day old female mosquitoes exposed to a fixed amount of insecticide for a fixed amount of time (WHO, 2018). This has led to concerns that bioassay results may not reflect mortality to insecticides deployed under more realistic field conditions and may have limited predictive value for operational decision-making (e,g, Grossman et al., 2020). Bioassay survival must therefore be converted to field survival to account for variable contact durations and other non-standardised factors in the field, and because insecticides select for mosquitoes capable of surviving exposure in the field and not for their ability to survive in a bioassay. Mosquito survival in bioassays has been found to be correlated with mortality in experimental huts (Churcher et al., 2016). To obtain estimates for the relationship between both as measures of survival, we therefore created a simple linear model to convert bioassay survival to survival in experimental huts which acts as our estimate for field survival to provide estimates for our mathematical model.

Equation 1b requires the conversion of the tracked bioassay survival (based on the Polygenic Resistance Score) to a field survival proportion. The relationship between bioassay survival and field survival was estimated using matched WHO cylinder bioassays with experimental hut trial data (data obtained from Churcher et al. 2016). In this study Churcher et al. used compared the link between bioassay mortality and experimental hut mortality for LLINs. For the quantitative genetics model the relationship between bioassay survival and experimental hut survival is needed, rather

than the relationship between mortalities. The obtained mortality data (both experimental hut and bioassay) was therefore converted to survival data. Generalised additive modelling was used to visually confirm the linear relationship. We used data obtained from by Churcher et al (2016) but restricted our model to only include the insecticide deltamethrin and *An. gambiae* s.l. mosquitoes, to reduce any confounding effects between different species responses to different insecticides. The total number of observations used was therefore 13. A simple linear model was then fit to the proportion surviving in the experimental hut against the proportion dying in the WHO cylinder:

$$K_i^F \sim \varphi_1 + \varphi_2 K_i^B$$

This can be used to estimate the relationship between bioassay survival and expected field survival. For the model, we use the estimate of 0.15 for the intercept and 0.48 for the regression coefficient, such that both values are rounded to two decimal places (Table S1). This means a fully susceptible population (with $\bar{z}$=0) would be expected to have an average of 15% survival to the insecticide in experimental huts. This seems intuitive as individuals vary both in their own, largely environmentally-determined, phenotypes that affect insecticide susceptibility (e.g., size, age, blood-feeding status) and the insecticidal environment they encounter i.e., concentration of insecticide (LLIN/IRS age) and duration of contact. Estimates are based on the relationship between paired female mosquito WHO cylinder bioassay survival and survival in experimental huts, and we assume this relationship also holds for male mosquitoes. We would like to highlight the lack of experimental hut studies which report male mosquitoes (i.e., numbers caught and mortality rates), which from an IRM perspective

makes parameterising models challenging as we are unclear on the level of selection applied to male mosquitoes.

Table S1: Linear Model of Field Survival and Bioassay Survival					
Parameter	Estimate	Upper 95% CI	Lower 95% CI	t value	P value
Intercept	0.14581	0.3649152	-0.07329093	1.465	0.1710
Bioassay Survival	0.48438	0.7949830	0.17376870	3.432	0.0056
Residual standard error: 0.1644 on 11 degrees of freedom<br>Multiple R-squared: 0.5171, Adjusted R-squared: 0.4732<br>F-statistic: 11.78 on 1 and 11 DF, p-value: 0.0056					

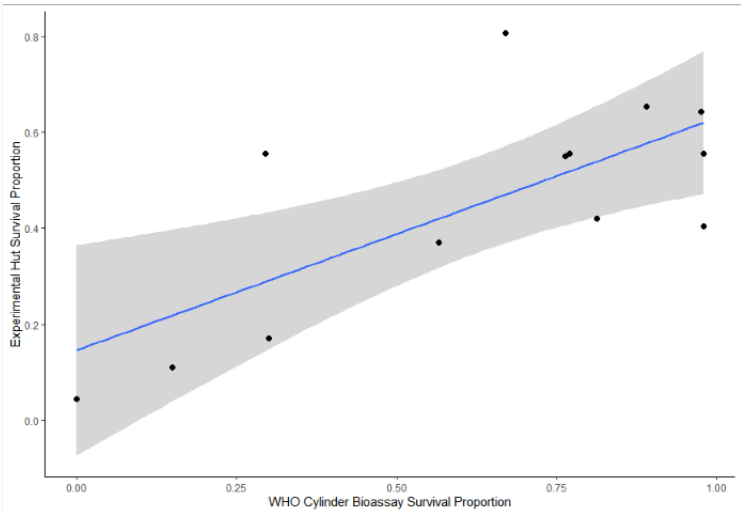

Figure S1 Relationship between experimental hut survival and bioassay survival. Regression line with 95% CI.

References

Churcher, T. S., Lissenden, N., Griffin, J. T., Worrall, E., & Ranson, H. (2016). The impact of pyrethroid resistance on the efficacy and effectiveness of bednets for malaria control in Africa. *ELife*, *5*(AUGUST), 1–26. https://doi.org/10.7554/eLife.16090

Grossman, M. K., Oliver, S. V., Brooke, B. D., & Thomas, M. B. (2020). Use of alternative bioassays to explore the impact of pyrethroid resistance on LLIN efficacy. *Parasites and Vectors*, *13*(1), 1–12. https://doi.org/10.1186/s13071-020-04055-9
