# Supplementary material for: Insecticide resistance management strategies for public health control of mosquitoes exhibiting polygenic resistance: A comparison of sequences, rotations, and mixtures
Source: Evol Appl. 2023 Apr 5;16(4):936–59. doi: 10.1111/eva.13546 (PMC10130562; doi:10.1111/eva.13546)
Supplement: Supplementary file 3 — Appendix S3 [file EVA-16-936-s001.pdf]

Supplement 3: Stability of the Polygenic Resistance Score with Population Standard Deviation

Table S2: Stability of the Polygenic Resistance Score with Population Standard Deviation					
	Population Standard Deviation				
Bioassay Survival (%)	0	1	5	10	25
0	0.000	0.000	0.000	0.000	0.000
5	47.368	47.350	47.367	47.468	48.507
10	100.000	100.012	99.944	99.897	100.263
20	225.000	225.020	224.971	225.285	225.776
50	900.000	900.011	900.019	900.460	901.115
