# Supplementary material for: Insecticide resistance management strategies for public health control of mosquitoes exhibiting polygenic resistance: A comparison of sequences, rotations, and mixtures
Source: Evol Appl. 2023 Apr 5;16(4):936–59. doi: 10.1111/eva.13546 (PMC10130562; doi:10.1111/eva.13546)
Supplement: Supplementary file 4 — Appendix S4 [file EVA-16-936-s002.pdf]

Supplement 4: Additional Analysis - Partial Rank Correlation and Generalised Linear Modelling

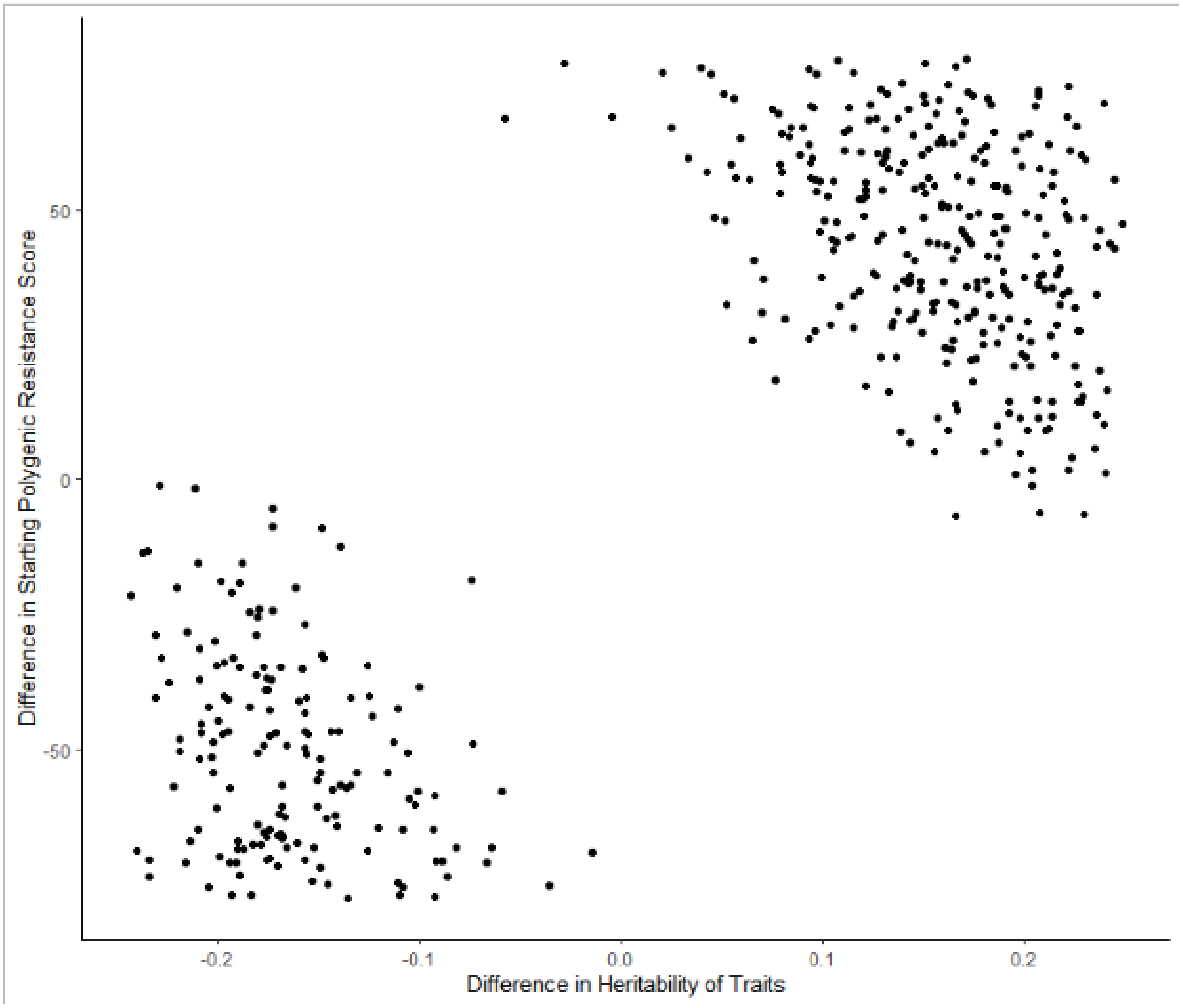

Figure S2 Explaining Scenarios where Sequences outperformed Mixtures. Highlights that for mixtures to be effective as an IRM strategy need to have broadly equal rates of evolution (equal heritability of traits) and broadly equal starting resistance values.

Partial Rank Correlation

Partial rank correlation (Figure S3) looks for correlations between the parameter input value and the outcome measure, in this case the strategy lifespan, and acts as a way of determining which parameters drive or inhibit the evolution of resistance. Increasing values of parameters with negative correlation drive the evolution of IR, while increasing the values of those with positive correlation slow the evolution of IR. The following parameters were identified as driving faster evolution of IR: male insecticide exposure, female insecticide exposure, intervention coverage and heritability. Fitness costs were associated with the slowing of the evolution of IR. Mosquito dispersal had no clear impact on the evolution of IR. These drivers of IR (as quantified by degree of correlation) were independent of cross resistance between insecticides.

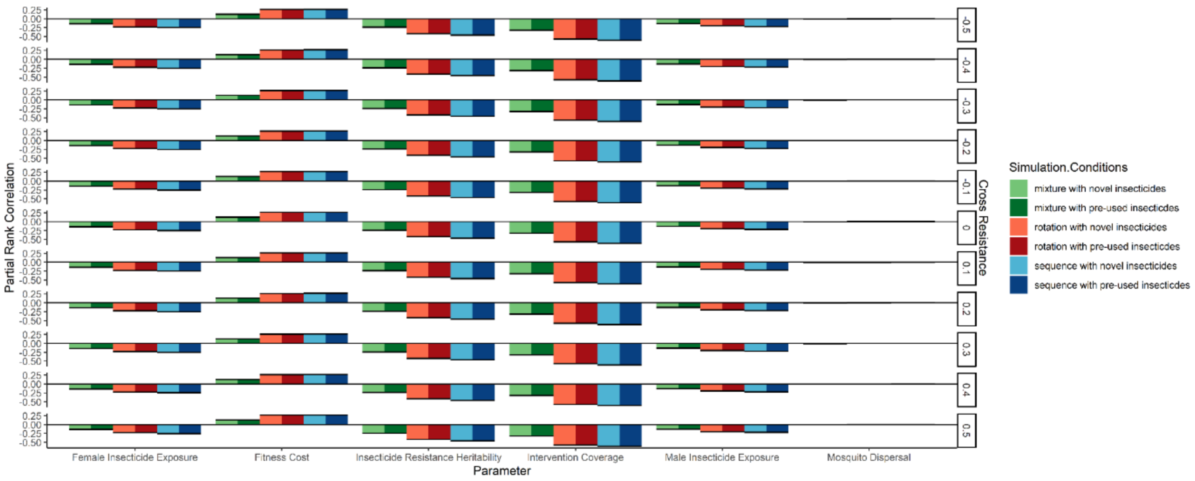

Figure S3 Partial Rank Correlation Between Parameter Inputs and Strategy Lifespan. Partial rank correlation was stratified by the IRM strategy (sequence [blues], rotation [reds], mixture [greens]), the starting polygenic resistance score of the simulation (novel = 0, pre-existing resistance = 50; darker colours indicate the simulations had pre-existing resistance. Each panel is the degree of cross resistance between the two insecticides. Error bars are the 95% confidence intervals of the

correlation estimate. Negative estimates are detrimental to IRM (decrease the longevity of the simulation), positive estimates are beneficial to IRM (increase the strategy lifespan).

Generalised Linear Modelling:

Negative estimates indicate the strategy lifespan decreases as the parameter value increases. Positive estimates indicate an increase in the strategy lifespan as the parameter value increases. Mosquito dispersal was found to have a complex non-linear relationship (Figure S3). Very low and very high rates of dispersal were found to slow the evolution of IR and prolong the strategy lifespans; however, all other rates of dispersal were found to increase the evolution of IR (i.e., to reduce the strategy lifespans). This non-linear relationship helps to explain the near zero correlation obtained for dispersal using partial rank correlation (Figure 4). Heritability was found to have the largest effect size on the strategy lifespan. The effect size differences between sequences and rotations were small when compared against the mixture baseline (Table 5).

Table 5: Negative Binomial Generalised Linear Model Output

Parameter	Estimate	Lower 95% CI	Upper 95% CI	Z value	p value
**Intercept**	10.0334360	10.02367741	10.0431955	2016.917	< 2e-16
Operational Parameters					
**IRM Strategy: Rotation (versus Mixture)**	-0.8650804	-0.86763721	-0.8625243	-664.958	0.0049746

**IRM Strategy: Sequence (versus Mixture)**	-0.8717067	-0.87426923	-0.8691447	-666.209	< 2e-16
**Intervention Coverage**	-1.8676578	-1.87229901	-1.8630168	-776.514	< 2e-16
Biological/Ecological Parameters					
**Heritability**	-4.8169772	-4.82884403	-4.8051110	-784.938	< 2e-16
**Fitness Cost**	2.9119299	2.89862874	2.9252317	426.938	< 2e-16
**Male Insecticide Exposure**	-0.5594688	-0.56188283	-0.5570550	-452.872	< 2e-16
**Female Insecticide Exposure**	-1.2946076	-1.29954112	-1.2896743	-511.900	< 2e-16
**Dispersal**	0.1572883	0.13245836	0.1821127	12.413	< 2e-16
**Dispersal Spline 1 at 0.238**	-0.1741744	-0.20152610	-0.1468182	-12.477	< 2e-16
**Dispersal Spline 2 at 0.785**	0.0820978	0.04635257	0.1178512	4.504	6.68e-06
**Starting Status: Pre-Used (vs Novel)**	-0.5586322	-0.56015723	-0.5571073	-721.346	< 2e-16
**Deployment Frequency 30 Generations (versus 10)**	0.0560758	0.05474972	0.0574019	82.884	< 2e-16
**Cross Resistance between insecticides**	-0.9361322	-0.93878331	-0.9334812	-684.026	< 2e-16
Null deviance: 1478816 on 172323 degrees of freedom<br>Residual deviance: 171869 on 172310 degrees of freedom<br>AIC: 1742112					

Number of Fisher Scoring iterations: 1

Theta: 63.125

Std. Err.: 0.265

2 x log-likelihood: -1742081.822

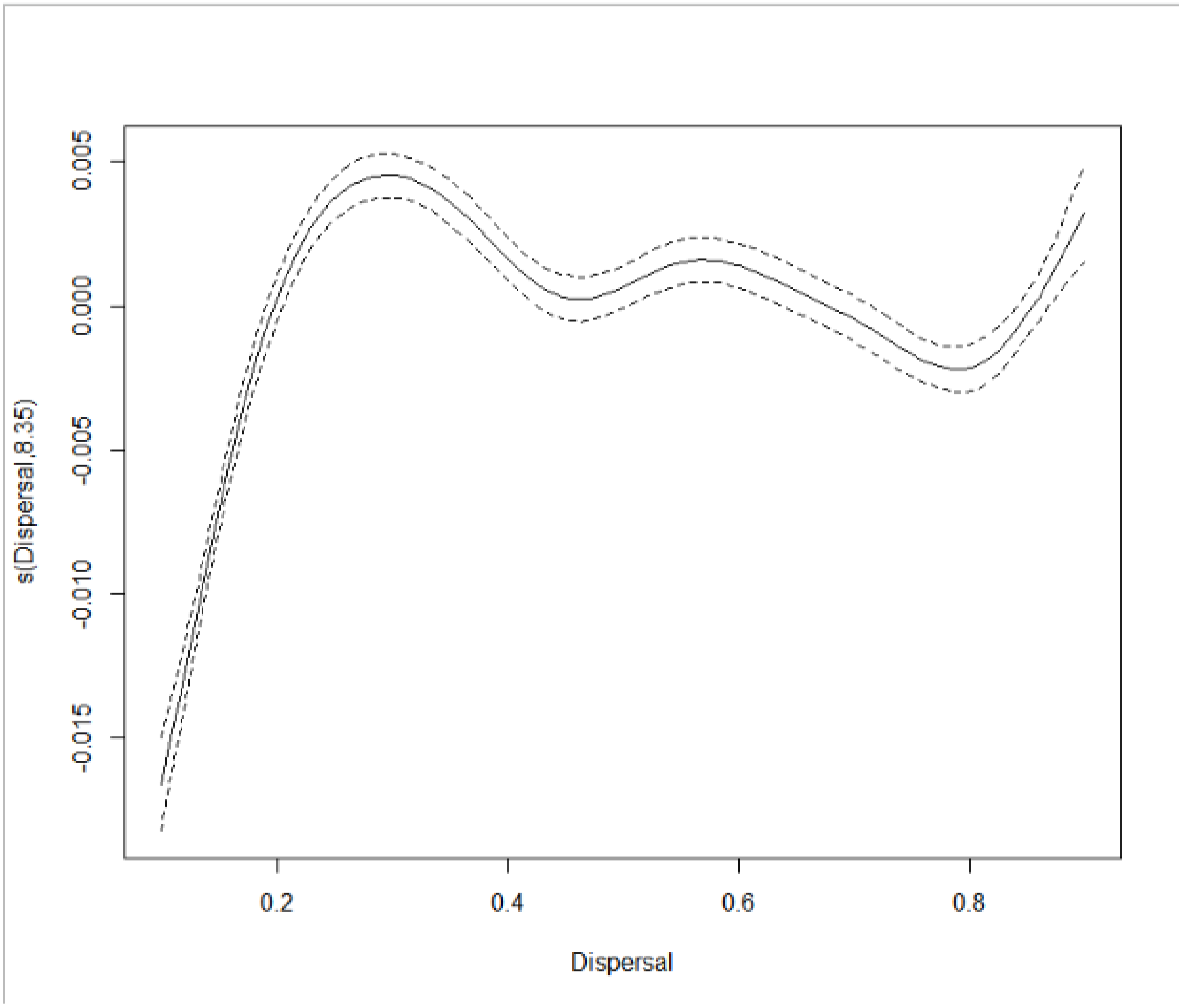

Figure S4 Generalised Additive Model smoothing over Dispersal to identify knot positions for piecewise GLM. Where the x-axis the dispersal rate, and the y-axis is the smoothing of the Dispersal parameter, and the effective degrees of freedom. High y-axis values indicate the simulation duration would be increased, while lower y-axis values indicate the simulation duration would be decreased. This plot was simply used to identify the knot positions of the dispersal parameter for the GLM. The knot position

of the splines was found through maximising the log-likelihood. Knot positions were calculated to a resolution of 3 decimal places.

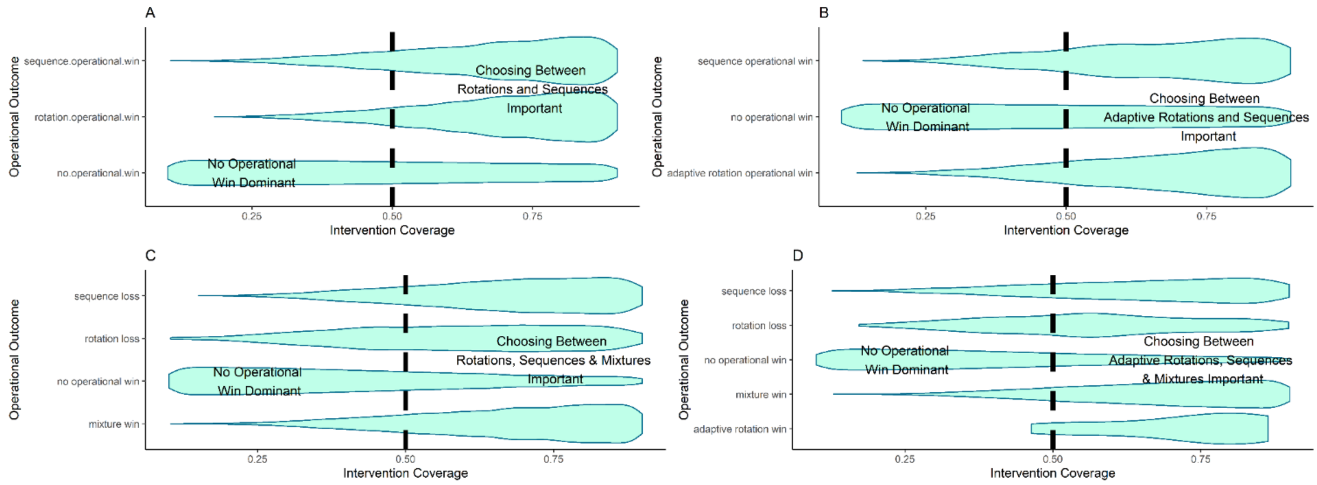

Figure S5 Violin Plot of the Operational Outcome in Relation to the Intervention Coverage. Panel A: Sequences versus Rotations – Equivalent Insecticides**.** Panel B: Sequences vs Adaptive Rotations – Unique Insecticides. Panel C: Sequences vs Rotations vs Mixtures – Equivalent Insecticides. Pane l D: Sequences vs Adaptative Rotations vs Mixtures – Unique Insecticides. Dotted line is 50% intervention coverage and indicates an approximate point where IRM strategy choice matters, which is the “no operational win” outcome is not dominant.
